# Therapeutic Vulnerabilities of the Key Genetic Drivers in Leiomyosarcoma

**DOI:** 10.3390/medsci14050571

**Published:** 2026-09-15

**Authors:** Ekaterina A. Lesovaya, Timur I. Fetisov, Beniamin Yu. Bokhyan, Varvara P. Maksimova, Evgeny P. Kulikov, Gennady A. Belitsky, Kirill I. Kirsanov, Marianna G. Yakubovskaya

**Affiliations:** 1 Laboratory of Molecular Mechanisms of Chemical Carcinogenesis, Institute of Experimental Oncology and Carcinogenesis, N.N. Blokhin Russian Cancer Research Center, Ministry of Health of Russia, 24 Kashirskoe Shosse, Moscow 115478, Russia; lesovenok@yandex.ru (E.A.L.); timkatryam@yandex.ru (T.I.F.); beniamin-bokhyan@mail.ru (B.Y.B.); lavarvar@gmail.com (V.P.M.); belitsga@mail.ru (G.A.B.); kkirsanov85@yandex.ru (K.I.K.); 2 Faculty of Oncology, I.P. Pavlov Ryazan State Medical University, Ministry of Health of Russia, 9 Vysokovol’tnaya St., Ryazan 390026, Russia; e.kulikov@rzgmu.ru; 3 Institute of Medicine, Peoples’ Friendship University of Russia, 6 Miklukho-Maklaya St., Moscow 117198, Russia

**Keywords:** leiomyosarcoma, genetic heterogeneity, genome instability, mutation, amplification, epigenetic changes, chromosomal translocation, signaling alteration, immunotherapy

## Abstract

Leiomyosarcoma (LMS) is a rare, aggressive soft-tissue sarcoma arising from smooth muscle cells. It has a high metastatic potential and limited therapeutic options. Despite advances in oncology, the molecular landscape of LMS remains incompletely understood, particularly regarding the genetic and epigenetic alterations that affect key signaling pathways. This review summarizes the current knowledge of mechanisms driving LMS pathogenesis, including somatic mutations in genes such as *TP53* and *RB1*, chromosomal instability, dysfunction of DNA damage response and repair, aberrant DNA methylation, histone modifications, and non-coding RNAs. Emerging treatment strategies include inhibitors of PI3K/AKT/mTOR and CDK4/6 signaling, epigenetic drugs, immunotherapy, and combinations of these approaches. However, the coexistence of multiple genetic abnormalities complicates diagnosis, prognosis, and therapeutic selection. Companion diagnostic tools that test candidate therapies ex vivo or in vitro may help exclude potentially ineffective targeted treatments.

## 1. Introduction

Leiomyosarcoma (LMS) is a sarcoma subtype with smooth muscle differentiation. It most commonly arises in the deep soft tissues of the extremities, retroperitoneum, or uterus, but it may also develop in large blood vessels, including the vena cava. In intramuscular and subcutaneous sites, LMS typically presents as an irregular, poorly circumscribed tumor with frequent central necrosis. Compared with other soft-tissue sarcoma (STS) subtypes, LMS has a higher metastatic rate (40–80% of cases) and a lower local recurrence rate. An exception to this is clinically indolent cutaneous LMS, or atypical intradermal smooth muscle neoplasm, which has a low metastatic potential and is generally managed with surgery alone. LMS arising at other sites usually requires multimodal treatment [1,2].

Histologically, LMS can be classified as a well-differentiated spindle-cell variant [3,4], either as a hypocellular myxoid variant with scant cytoplasm and nuclei of oval, spindle or stellate morphology [5] or a hypercellular epithelioid variant with eosinophilic cytoplasm and cytologic atypia [3,4,6]. Its smooth muscle origin is reflected by immunohistochemical markers, such as α-smooth muscle actin (α-SMA), vimentin, desmin, h-caldesmon, and histone deacetylase 8 (HDAC8) [5,7,8]. Poorly differentiated LMS may lose smooth muscle marker expression and can therefore be misdiagnosed as undifferentiated pleomorphic sarcoma [3,5,6,7]. In uterine LMS, the distinction from benign leiomyoma is particularly important because the two entities share clinical and morphologic features; however, leiomyoma has fewer copy-number variants and somatic single-nucleotide variants [9].

Surgery remains the cornerstone of LMS treatment and may be combined with adjuvant or neoadjuvant radiotherapy and doxorubicin- or ifosfamide-based chemotherapy [10]. Conventional cytotoxic agents, including doxorubicin, ifosfamide, dacarbazine, docetaxel, and gemcitabine, are used primarily for advanced disease [6,7]. The minor-groove binder trabectedin has reached phase III trials and has shown efficacy in combination regimens with a favorable safety profile [6,7]. Nevertheless, new druggable targets are needed. This review therefore examines the genetic and epigenetic abnormalities in LMS and considers how they may inform treatment optimization.

## 2. Complex Genetics of LMS

LMS has a highly complex genetic landscape. Recurrent somatic mutations, copy-number gains, and deletions affect *TP53*, MED12, ATRX, MEN1, IGF1R, *MYOCD*, and *PTEN* [6,11,12,13,14,15,16,17,18,19,20,21,22,23,24]. Next-generation sequencing (NGS), including RNA, whole-exome, and whole-genome sequencing, has identified recurrent gains at 1p, 1q, 2p, 3p, 6p, 8q, 10q, and 18q and losses at 2q, 4q, 6p, 6q, 7p, 7q, 13q, 14p, 16q, 19p, Xp, and Xq [11,14,17,25,26,27]. Common abnormalities involve *RB1* (13q14.3–q21.1), *PTEN* (10q11–21.2), *TP53* (17p13), and ATRX/DAXX (Xq21.1/6p21.32), as well as copy-number gains at 5p15.33 (TERT), 8q24.21 (C-MYC), and 17p11.2 (*MYOCD*, MAP2K4) [11,12,15,19,20,28,29,30,31]. Additional heterogeneous alterations affect genes involved in DNA damage response and telomere maintenance (TOPORS, ATR, TP53BP1, TOP2A, TELO2); cell-cycle regulation and apoptosis (PSDM11, CASP7, XPO1); epigenetic silencing and transcriptional reactivation (HIST3H3, SETD7, KMT2C); MAPK signaling and muscle-cell proliferation (MAPK14, DUSP10, MEF2C); mRNA stability (ZFP36L1, SRSF5); differentiation and metabolism through Notch, Hedgehog, and PI3K/AKT signaling (MTOR, LAMA4, NKX6-1); and survival and stress adaptation (ERK5, YWHAE, VIPR2) [12,32,33,34,35,36].

### 2.1. Gene Abnormalities Associated with Genome Instability and Immune Escape

*RB1* aberrations occur in approximately 30% of LMS and can truncate the *RB1* protein, resulting in the loss of function [11,14,15]. For example, *RB1* loss converts poorly spherogenic Trp53-null LMS cells into highly spherogenic cells [37]. More broadly, the *RB1*–cyclin D1 pathway may be disrupted by alterations in *RB1*, CCND1, CCND3, CDK4, PIK3CA, and CDKN2A [17,20,26,27,38]. Germline *TP53* alterations often co-occur with somatic *RB1* alterations, and the converse pattern has also been reported [39,40].

LMS is also associated with hereditary cancer predisposition syndromes. Li–Fraumeni syndrome, caused by germline *TP53* mutations, confers susceptibility to multiple malignancies, including LMS [39,40]. Rectal LMS and primary axillary vein LMS have been reported as initial manifestations of Li–Fraumeni or Li–Fraumeni-like syndromes [41,42]. Similarly, patients with hereditary retinoblastoma caused by *RB1* loss may develop visceral or bladder tumors [43], and several female survivors have developed uterine LMS [44,45]. Intracranial LMS arising after glioblastoma has also been described, although the specific genetic abnormalities were not reported [46].

Genomic instability in LMS reflects not only loss of the key genome-integrity regulators *RB1* and *TP53* but also alterations in other related gene families. Characteristic features include deficient DNA damage response (DDR) and repair, low tumor mutational burden (TMB), and low microsatellite instability (MSI). The DDR is a signal-amplification cascade that detects DNA damage, induces cell-cycle arrest, and initiates repair. Its major repair mechanisms include homologous recombination (HR), mismatch repair (MMR), base-excision repair (BER), and nucleotide-excision repair (NER) [47,48].

Genes encoding mismatch-repair regulators are frequently altered in LMS. In a study of 30 patients with sarcoma, germline mutations were most common in MSH2 (50%) [49]. HR pathway dysregulation involves alterations in *PTEN*, BRCA2, ATM, CHEK1, XRCC3, CHEK2, BRCA1, RAD51, and FANCA [6,12,50,51,52]. DDR gene alterations were identified in 24% of 165 uterine LMS samples and 10% of 125 non-uterine LMS samples [53,54,55].

Alterations that affect tumor immunogenicity may further contribute to immune escape. Analysis of The Cancer Genome Atlas (TCGA) identified the amplification of the RNA-editing genes ADAR, ADARB1, and ADARB2 in 15% of LMS cases, suggesting a role in establishing an immunologically cold phenotype [56]. Consistent with this possibility, CTHRC1, which encodes a regulator of vascular remodeling, was overexpressed in 80% of uterine LMS samples and correlated with poor survival [57]. CTHRC1 also recruits M2 macrophages, thereby promoting a microenvironment favorable to tumor progression [58].

### 2.2. Other Gene Mutations and Amplifications

Beyond these recurrent tumor-suppressor alterations, several other abnormalities have potential clinical relevance. The cell-cycle regulators PLK1 and CHEK1 are frequently overexpressed in uterine LMS [59]. In an immunohistochemical study of tumors from 60 patients, the overexpression of CDK8 was associated with poor prognosis [60]. STMN1, which encodes a microtubule-destabilizing cell-cycle regulator, was overexpressed in 40% of uterine LMS cases [20]. Progesterone receptor (PR) expression can identify patients as having longer survival [61], and hormone therapies targeting estrogen and progesterone receptors have been used particularly in uterine LMS [62,63,64]. The screening of 48 receptor tyrosine kinase genes also identified ROR2 overexpression in LMS [65]. The epigenetic regulators EZH2 and BRD9 were likewise overexpressed [66,67,68], and high EZH2 and nicotinamide N-methyltransferase (NNMT) expression may help distinguish uterine LMS from benign leiomyoma preoperatively [69].

Growth factors have also been reported to be affected in LMS. Metastatic LMS was characterized by FGFR1 and PDGF amplifications [70,71]. Insulin-like growth factor (IGF-II) mRNA-binding protein 3 (IMP3) was reported to be positive in 50% uterine LMS while being completely absent in normal endometrial tissue [20]. NGS analysis of LMS samples from 10 patients showed a chromosomal rearrangement involving FGFR1-TACC1 and a copy-number loss for MTAP, CDKN2A, and CDKN2B using array comparative genomic hybridization [72].

### 2.3. LMS-Specific Fusions

Unlike synovial sarcoma and well-differentiated liposarcoma, LMS has no single characteristic gene fusion. But while its fusion landscape is heterogeneous, several recurrent or potentially actionable chimeric genes have been reported. One group involves epigenetic regulators, including KDM2B-CREBBP, ZC3H7-BCOR, JAZF1-BCORL1, EPC1-BCOR, LPP-BCOR, and BCOR internal tandem duplication [68]. KAT6B/A–KANSL1 fusions, involving a histone acetyltransferase and its co-activator, were reported in nine uterine LMS cases [28,73].

ALK fusions have been reported in a subset of LMS, including fusions with the microtubule regulator KANK2, as well as TNS1-ALK and ACTG2-ALK chimeric proteins [74,75].

NTRK family genes encode neurotrophic tyrosine kinases and are reported to be found in LMS. A single case of LMNA-NTRK1 fusion-positive LMS has been recently described [76]. An STRN3-NTRK3 fusion was found in a patient with bone LMS and was shown to play a role in malignant transformation [77]. NTRK3-DEFB132, TPM3-NTRK1, LMNA-NTRK1, RBPMS-NTRK3, and TPR-NTRK1 fusions have also been described [78,79].

NR4A3-rearranged LMS is characterized by a chimeric NR4A3 transcription factor that modulates downstream gene expression and participates in proliferation, apoptosis, and differentiation. Its most frequent fusion partners are PGR, CARMN, ACTB, and SLCO5A1 [80,81,82].

A novel MAN1A1-ROS1 fusion, involving the receptor tyrosine kinase ROS1 and an enzyme involved in oligosaccharide biosynthesis, was described in a patient with thigh LMS [83].

An EEF1A1 fusion has also been reported in uterine LMS. EEF1A1 participates in translation by delivering aminoacylated tRNAs to the ribosomal A site [84], and EEF1A1 rearrangements have been associated with activation of EGFR and FLT3 signaling [57].

PLAG1 rearrangements were initially found in the myxoid uterine [80,85]. Recently, five cases of LMS were reported to harbor PLAG1 fusions (TRPS1-PLAG1, RAD51B-PLAG1, and TRIM13-PLAG1). A novel MIR143HG-PLAG1 fusion was found in one case of rectal LMS [86,87,88].

### 2.4. Molecular Features of LMS Subtypes

Recent molecular studies have classified LMS into three subtypes with distinct genetic and transcriptional features [26,89,90,91]. Subtype I, well-differentiated soft-tissue LMS, expresses muscle-associated genes. Subtype II, dedifferentiated soft-tissue LMS, shows reduced expression of these genes and a less differentiated phenotype. Subtype III is largely uterus-specific [89]. More than 5000 genes are differentially expressed across these subtypes; their principal characteristics are summarized in Table 1.

Primary and metastatic LMS also have distinct expression profiles. Primary tumors show increased expressions of OSTN, NLGN4X, NLGN1, SLITRK4, MASP1, XRN2, ASS1, RORB, HRASLS, and TSPAN7, whereas metastases show increased expressions of TNNT1, FOLR3, TDO2, CRYM, GJA1, TSPAN10, THBS1, SGK1, SHMT1, EGR2, and AGT. These differences may provide additional biomarkers of progression [96].

### 2.5. Circulating Tumor DNA as a Prognostic Factor for LMS

Circulating tumor DNA (ctDNA) provides a rapid, non-invasive source of material for NGS and may support LMS diagnosis, treatment selection, and recurrence monitoring [97,98]. In patients with metastatic LMS, ctDNA analyses have detected one or more cancer-associated alterations, including changes in *TP53*, BRAF, CCNE, EGFR, PIK3CA, FGFR1, *RB1*, KIT, *PDGFRA*, RAF1, ERBB2, MET, *PTEN*, TERT, FGFR4, APC, and NOTCH1 [99,100]. A clinical trial evaluating a biomarker protocol for localized LMS is ongoing [48].

## 3. Epigenetic Features of LMS

Epigenetic alterations complement the genomic abnormalities described above and may help explain LMS development, progression, and therapeutic response. Although this field remains evolving and several findings require further validation, the principal epigenetic mechanisms implicated in LMS are summarized below.

### 3.1. DNA Methylation

In LMS, multiple genes are inactivated through promoter or enhancer hypermethylation. Reported targets include genes encoding chromatin-modifying enzymes (KAT6A, KMT2A, EZH2) and chromatin- or DNA-binding proteins and regulators (CTNNB1, PBX3, SATB1, MEIS, ALX1, CBLN1, CORIN, MGMT, DUSP6, FOXP1, GATA2, IGLON5, NPTX2, NTRK2, STEAP4, PART1, PRL, and COMMD1-BMI1) [7,101,102]. Uterine and non-uterine LMS also differ in their DNA methylation profiles. In particular, progesterone receptor DNA hypermethylation is more prominent in uterine LMS than in extrauterine tumors [101].

### 3.2. Histone Modifications

Histone modifications include methylation, acetylation, phosphorylation, ubiquitination, and SUMOylation, which are controlled by histone acetyltransferases and deacetylases (HATs/HDACs), methyltransferases and demethylases (HMTs/HDMs), and other related enzymes [103,104]. Dysregulation of these enzymes has been documented in LMS. HDAC’s overexpression may contribute to pathogenesis, and HDAC4 has been associated with poor prognosis [105]. The suppressor of variegation 3–9 homolog 2 (SUV39H2), a histone methyltransferase that promotes double-strand break repair by recruiting phosphorylated histone H2AX (γH2AX), is upregulated in LMS. Both SUV39H2 and γH2AX may serve as prognostic biomarkers and therapeutic targets [106,107].

### 3.3. Non-Coding RNA

Multiple miRNAs were found to be differentially expressed in LMS and exerted both pro-cancer and anti-tumor effects. In the study of the Sarcoma miRNA Expression Database using the Enrichr analysis tool, the expression of 301 miRNAs was found to be significantly different between LMS and healthy smooth muscle samples [108]. In particular, the overexpression of miRNA-654-3p, miRNA-181b, miRNA-34a, and miRNA-369-3p was found in LMS cell cultures and in patient samples; an anti-oncogenic role was found for these miRNAs [20,109,110]. MiRNAs belonging to the let-7 family and associated with HMGA regulation (let-7 repression leads to HMGA overexpression) were downregulated in LMS, apparently acting as a tumor suppressor in smooth muscle tissue [111,112]. It was demonstrated by an miRNA-sequencing-based approach that the downregulation of miR-10b-5p stimulated the proliferation of uterine LMS cells, and miR-10b-5p could also serve as a tumor suppressor [113]. The tumor-suppressive effects of miRNA-1 and its downregulation in highly malignant LMS cells were demonstrated in vitro [114]. In the LMS samples from patients, the downregulation of five MDM2-targeting miRNAs— miR-223, miR-379, miR-377, miR-495, and miR-758—as well as two CDK4-targeting miRNAs—miR-486-5p and miR-539—was demonstrated, suggesting the anti-tumor role for this miRNA pool [115]. MiRNA-200c limits epithelial–mesenchymal transition (EMT), migration, and invasion through the inhibition of ZEB1/2, VEGFA, and TIMP2 genes [116,117].

MiRNA-130b was found to be overexpressed in LMS and is associated with invasion, migration, and metastasis [118]. The dysregulation of miR-148a-3p, 27b-3p, 124-3p, 183-5p, and 135b-5p expression was associated with tumor relapse, increased metastasis, and poor survival rates in uterine LMS patients, but their specific roles need further elucidation [119]. Table 2 summarizes the miRNAs involved in LMS pathogenesis.

Evidence regarding long non-coding RNAs (lncRNAs) in LMS remains limited. In vitro, EGFR-AS1 increased PD-L1 expression through the EGFR/MYC pathway, reduced T-cell infiltration, and promoted immune escape [121].

### 3.4. RNA Methylation

Approximately 170 RNA modifications have been described. The most prevalent, N6-methyladenosine (m6A), regulates multiple biological processes, including carcinogenesis and immune responses [7]. In LMS, the m6A regulators METTL3, METTL14, WTAP, FTO, ALKBH5, and YTHDF1–3 have been associated with TNFα, KRAS, c-MYC, and PD-L1 expression or activity [122]. These observations identify RNA methylation as another potential therapeutic axis; in particular, FTO inhibition has been proposed as a treatment strategy [123].

## 4. Changes in Signaling and Therapeutic Approaches

### 4.1. DNA Damage and Repair Pathways

Genetic abnormalities in DDR components, including ATRX, FANCA, and BRCA1/2, translate into therapeutically relevant defects in DNA repair signaling [124]. This creates opportunities for targeted treatment, several of which have entered preclinical or clinical evaluation. The ATR inhibitor elimusertib (BAY1895344), for example, showed in vivo activity in an ATRX-mutant uterine LMS model [125]. BRCA1/2 inactivation, reported in 10–50% of LMS, provides a rationale for PARP inhibition. Olaparib, alone or with cisplatin, showed activity in a preclinical uterine LMS model with homologous recombination deficiency (HRD) [51]. Current clinical evidence includes a phase II study of temozolomide plus olaparib as a fourth-line therapy for uterine LMS and case reports of olaparib activity in tumors with mutated DDR genes [126,127,128]. However, despite the encouraging results of phase II clinical trials, further studies have shown that this combination does not provide a significant advantage to patients in recurrence-free survival compared to classical therapy in both uterine sarcoma and soft-tissue sarcomas [10.1200/JCO.2025.43.16_suppl.11507; 41354211]. Nevertheless, the combination of olaparib and trabectedin is considered a promising direction in therapy, and researchers are currently focusing on the search for markers predicting response to therapy, as longer recurrence-free survival was observed in patients with a high PARP-1 expression, which, according to the researchers, positions PARP-1 to be considered a promising marker of response [41354211].

### 4.2. Hedgehog Pathway

Hedgehog signaling is activated in uterine LMS and may represent another therapeutic vulnerability. In vivo studies have shown anti-tumor activity of the SMO inhibitor LDE225 and the GLI inhibitor GANT61 [129]. In vitro treatment with the DNA methyltransferase inhibitor 5-aza-dC also reduced GLI and SMO expression, thereby inhibiting LMS cell proliferation, migration, and invasion [130]. Several SMO inhibitors are already FDA-approved for other indications, supporting further evaluation in LMS [7].

### 4.3. VEGFR

Aberrant VEGF signaling supports LMS angiogenesis and growth and is therefore a potential therapeutic target [131]. In vitro, the tyrosine kinase inhibitor PTK787 suppressed VEGF-A expression and increased LMS cell death [132]. Endoglin, a regulator of invasion and VEGF secretion, is associated with poor prognosis; inhibiting its expression or activity may offer another antiangiogenic strategy [133]. Colony-stimulating factor 1 (CSF1), which may promote angiogenesis by recruiting macrophages, was highly expressed in 149 primary non-gynecologic LMS cases and also represents a potential target [134].

Clinical studies have begun to test angiogenesis inhibitors in combination regimens. A phase I/II trial of bevacizumab plus the histone deacetylase inhibitor valproic acid produced partial responses in patients with LMS [135]. In a phase II trial, axitinib combined with the immune checkpoint inhibitor pembrolizumab showed manageable toxicity and preliminary activity in advanced sarcomas, including LMS [131].

### 4.4. Inflammation and Immune Response Signaling

Inflammatory signaling also contributes to LMS pathogenesis. Orosomucoid 1 (ORM1), an acute-phase protein, was increased in uterine LMS and showed positive immunohistochemical staining [136]. Consistent with this finding, CD163-positive macrophages induced IL-6 signaling and promoted tumor development and progression in vitro and in vivo [137]. Dysregulated TGF-β, angiogenesis, and macrophage migration inhibitory factor pathways may therefore provide targets for combination immunotherapy [138]. Crosstalk between JAK/STAT activation and PD-L1 and IDO1 expression has likewise been demonstrated in LMS cells and patient samples [139]. The inflammatory phenotype of LMS cells, the activation of immune response due to frequent mutational events, and the high levels of T-cell-related gene expression in LMS, particularly the pleiomorphic LMS subtype, could suggest LMS as a candidate for targeted immunotherapy similar to undifferentiated pleiomorphic sarcoma (UPS) [140,141,142]. Currently, the efficacy of immune checkpoint inhibitors (ICIs) in leiomyosarcoma (LMS), both as a monotherapy and in combination with other agents, is being actively studied [143,144,145,146,147,148,149,150,151,152,153,154,155]. However, there is still no consensus on their effectiveness. Thus, limited efficacy has been observed in several studies. For instance, in a case report by Ishihara S. et al., a response of leiomyosarcoma to nivolumab after radiation therapy was described [144]. In studies of advanced LMS, the ORR of ICI combinations with gemcitabine, cediranib, and regorafenib ranged from 15% to 20%, with PFS around 4–5 months [145,150,152]. Meanwhile, nivolumab monotherapy showed no objective responses, and the median PFS was only 1.8 months in advanced leiomyosarcomas [143]. In addition, combinations of ICIs with mTOR and IDO1 inhibitors also proved ineffective [146,147].

Other antigens on immune cells (CD70, CD47) have been considered as targets for the therapy in preclinical studies, but they have not yielded any clinical trials to date [156,157].

However, the resistance that develops by upregulating alternative immune checkpoints, such as TIM-3 and LAG-3, as well as by the loss of *PTEN*, has also been reported, probably explaining the variable responsiveness to checkpoint inhibitors [158,159]. Single-cell mapping of uterine LMS revealed an extremely immunosuppressed tumor microenvironment dominated by depleted CD8+T cells, M2 macrophages and N2 neutrophils [138]. A clinical trial of immunotherapy with the single-agent nivolumab in advanced-stage LMS showed no clinical benefit, probably due to the immunosuppressive tumor microenvironment [160].

In addition to pharmaceutical immunotherapy, LMS is not commonly characterized by the expression of cancer-testis antigens (CTAs), which could potentially be targeted by T-cell receptor (TCR) gene therapy. Notably, a preclinical study has been reported in which MAGE-A4 TCR-engineered T cells, recognizing epitopes in MAGE-A4, could represent another potential immunotherapeutic approach for LMS [161].

Immune checkpoint inhibitors have demonstrated limited efficacy in LMS treatment due to the low tumor mutation burden (TMB), low microsatellite instability (MSI), heterogeneous immunosuppressive microenvironment, downregulation of antigen presentation pathways, and upregulation of alternative immune checkpoint pathways, as well as epigenetic alterations affecting immune response. Combination strategies are being explored to overcome these limitations, including immune checkpoint inhibitors with chemotherapy, targeted therapies with immunomodulators, radiation therapy with immunotherapy, the combination of epigenetic modifiers with immunotherapy, and targeting immune suppressive pathways in the tumor microenvironment.

### 4.5. Proliferative Signaling (Growth Factors and Cell-Cycle Regulators)

The diverse genetic abnormalities in LMS converge on multiple kinase cascades. Because kinase inhibitors have been used in oncology for more than two decades, an expanding library of compounds is available for evaluation in this genetically complex disease. These agents are promising candidates for targeted therapy, although most evidence remains preclinical.

More specifically, uterine LMS cells harboring MAP2K4 amplification demonstrated tumor growth inhibition of LMS xenografts in vivo after the treatment with PLX8725, a novel MAP2K4 inhibitor [162]. TYRO3 (TYRO3 protein tyrosine kinase) and AXL (AXL receptor tyrosine kinase) activation in LMS cells has made these molecules new potential targets in many oncological diseases, including LMS [163]. In vivo studies have suggested that blocking HGF/SF:c-Met signaling provides a rationale for the development of HGF/SF:c-Met inhibitors [164]. Acrogranin, an acrosomal cysteine-rich glycoprotein that is the precursor of growth-modulating peptides, was reported to be overexpressed in LMS. Moreover, the exogenic overexpression of acrogranin led to the malignant transformation of LMS cells in the in vitro experiments [165]. HAI-1 (hepatocyte growth factor activator inhibitor type 1) and HAI-2 (hepatocyte growth factor activator inhibitor type 2) were demonstrated to act as possible tumor suppressor genes for uterine LMS. They could be considered therapeutic agents for the treatment of LMS, but no preclinical or clinical study has been reported [166]. The PLK4 (Polo-like kinase 4) inhibitor CFI-400945, individually or in combination with the ATM (ataxia telangiectasia mutated) inhibitor AZD0156, was studied in vitro and in vivo; preliminary anti-tumor activity was reported [167]. One clinical case report has been described for ALK inhibitors, in which ALK inhibitors alectinib and lorlatinib induced a 16-month response to the second generation of therapy [168].

### 4.6. Wnt/β-Catenin

Wnt/β-catenin signaling provides a further link between molecular dysregulation and potential treatment. β-Catenin, a central WNT pathway component and regulator of cell adhesion, is upregulated in different cellular compartments in more than 25% of LMS cases, and it is particularly prominent in high-grade tumors [169]. The transient receptor potential vanilloid 4 (TRPV4) channel, an indirect regulator of WNT/β-catenin signaling, is also upregulated and may represent a therapeutic target [170,171]. Further work is needed to define the clinical relevance of this pathway.

### 4.7. PI3K/Akt/mTOR Signaling

PI3K/AKT/mTOR signaling promotes LMS cell proliferation and is a plausible therapeutic target. An increased expression of the mTOR targets pS6RP and p4EBP1 correlates with poor prognosis [172]. The dual PI3K/mTOR inhibitor BEZ235 impairs LMS growth in vitro and in vivo [173]. Rapamycin/sirolimus inhibits uterine LMS growth in vitro, although its in vivo effect is primarily cytostatic. Combining rapamycin with the Aurora kinase inhibitor MLN8237 produces synergistic inhibition in vitro and in vivo [172]. Natural compounds have also been evaluated: curcumin suppresses uterine LMS xenograft growth by inhibiting AKT/mTOR signaling and inducing autophagy and apoptosis [174,175], whereas epigallocatechin-3-gallate enhances curcumin uptake and lowers the concentration required for pathway inhibition [174,176].

PI3K/mTOR activation may also promote immune escape, providing a rationale for combining pathway inhibitors with immune checkpoint blockade [160]. Clinical activity has nevertheless been modest. In the phase I/II SARC023 study of sirolimus plus ganetespib, one patient with LMS achieved a partial response [177]. In a phase II trial, the PI3KCB inhibitor GSK2636771 showed modest single-agent activity in relapsed LMS with *PTEN* inactivation or deletion [178].

A phase III trial examining the role of ridaforolimus, another mTOR inhibitor, on over 700 patients with metastatic sarcoma, of which 231 had LMS, showed only modest improvements in PFS compared with the placebo, and had no effect on OS [179,180]. In a dose-escalation study of sapanisertib (CB-228/TAK-228), a selective ATP-competitive dual mTORC1/2 inhibitor, in combination with metformin, its safety, tolerability, and early clinical activity was demonstrated in two patients with LMS [181].

### 4.8. Notch Signaling

Notch signaling presents another option for the targeted therapy of LMS. It was shown in vitro that Notch proteins were differentially expressed in LMS; the overexpression of HES1, a downstream effector of Notch, was also demonstrated. The Notch inhibitors DAPT and MK-0752 decreased the expression of HES1 and decreased LMS cells [182]. A preclinical study has demonstrated a cooperative role for Pten and Tp53 suppression in complex karyotype sarcomas like LMS, and Notch was considered a regulator in the cross talk of these pathways during tumor progression. [183]. A first-in-human phase I study of the Notch inhibitor LY900009 in advanced LMS was completed. Several side effects were recorded, and the recommended minimally tolerated dose was calculated for further trials [184]. Another phase I, open-label, multi-center, nonrandomized dose-escalation study of the Notch inhibitor LY3039478 allowed the development of doses and regimens for a phase II clinical trial. Clinical activity (tumor necrosis, metabolic response, or tumor shrinkage) was observed in patients with breast cancer, LMS, and adenoid cystic carcinoma [185].

### 4.9. Other Signaling Alterations

The genetic abnormalities described above lead to multiple other changes in signaling, but not every aberrant protein yielded a therapeutic target with the development of novel drugs. Several dysregulated proteins and other macromolecules are currently considered as potential prognostic factors. More specifically, Hippo signaling was found to be dysregulated in more than 50% cases of LMS, suggesting it as a positive prognostic factor [186]. Myostatin, a negative regulator of muscle tissue growth, could be used as a biomarker to distinguish pleomorphic LMS from other high-grade sarcoma subtypes [187]. An elevated serum level of triglycerides was associated with an increased risk of LMS [188].

Separate proteins could serve not only as prognostic markers but also as markers of chemosensitivity. Bcl-2 proteins contribute to intrinsic LMS chemoresistance by increased expression of antiapoptotic genes/proteins and inhibition of proapoptotic BH3 mimetic proteins [189]. Human equilibrative nucleoside transporter 1 (hENT1), the major gemcitabine transporter into cells, are associated with gemcitabine efficacy both in patients with advanced LMS [190]. In an in vitro study on uterine LMS cells, it was demonstrated that microtubule inhibitor eribulin suppressed the expression of microtubule regulator stathmin. Moreover, stathmin overexpression enhanced the antiproliferative effect of eribulin [191]. Additionally, β-tubulin TUBB3 overexpression led to the eribulin resistance in LMS cells, and siRNA knockdown of TUBB3 restored eribulin sensitivity [192]. In a nonrandomized, multi-center phase II clinical trial, the eribulin–gemcitabine combination therapy showed anti-cancer activity and a favorable safety profile in LMS [193]. In addition, the efficacy and safety of eribulin, in combination with Lenvatinib, were demonstrated in a multi-center phase Ib/II study for advanced LMS [194].

Some other targets have been investigated in preclinical studies. The expression of latent membrane protein 2 (LMP2)-induced calponin h1, an actin-binding protein regulating smooth muscle function, plays a role in LMS pathogenesis; therefore, LMP2 may be a tumor suppressor in LMS [195,196]. In silico and in vitro investigations revealed the proapoptotic activity of the novel DNA minor groove binder NSC-260594/XMH95 in LMS cells by inducing the expression of PMAP1/NOXA, BIK, HRK and BBC3/PUMA [197]. The therapeutic potential of the RNA polymerase I transcription inhibitor, CX-5461, was demonstrated in the experiment in vitro. The mechanism of the anti-cancer effect was associated with cell-cycle arrest and reduced ribosomal DNA transcription [198]. Preclinical studies in vitro have demonstrated the therapeutic potential of the combinations of the gamma secretase inhibitor MK-0752 and doxorubicin, MK-0752 and docetaxel, and MK-0752 with gemcitabine and docetaxel [199].

Modulators of nuclear receptors present a separate class of potential pharmaceuticals for LMS therapy. The peroxisome proliferator-activated receptor γ (PPARγ) agonists, thiazolidinediones—for example, pioglitazone—revealed its inhibitory effects on LMS cell survival in vitro, as well as its stimulation of apoptosis induction [200]. Ulipristal acetate (UPA), a selective progesterone receptor modulator that is used for the treatment of benign leiomyoma, revealed anti-cancer STAT3/CCL2-dependent effects in models of uterine LMS in vitro and in vivo [201]. A promising approach to LMS therapy could be melatonin treatment, as tumor regression was demonstrated in a patient-derived LMS xenograft study [202,203].

### 4.10. Epigenetic Regulators in Cell Signaling

The preceding sections have shown that genomic and epigenetic alterations in LMS are closely interconnected. Accordingly, dysregulated epigenetic regulators are themselves potential therapeutic targets. BET family proteins, including BRD2, BRD3, BRD4, and BRD9, are overexpressed in uterine LMS [67,68]. In preclinical studies, the BET inhibitors JQ1, TP-472, and I-BET762 suppressed LMS cell proliferation through cell-cycle arrest and modulation of Hedgehog and EMT signaling [67,68,204]. LMS models have also shown aberrant upregulation of class I HDAC proteins [205]. The HDAC inhibitor quisinostat demonstrated strong preclinical activity and synergy with doxorubicin [206]. DNA methyltransferase inhibitors, including azacitidine, 5-aza-2′-deoxycytidine, and guadecitabine, significantly suppressed LMS growth in vitro and in vivo [207]. In murine uterine LMS xenografts, the cardiac glycosides proscillaridin A and lanatoside C enhanced the activity of DNMT and HDAC inhibitors [208].

Epigenetic abnormalities other than DNA/histone modifications could also represent targets for the development of novel therapies. For example, in a preclinical study, the anti-tumor activity of the SUMOylation inhibitor 2′,3′,4′-trihydroxyflavone (2-D08) was demonstrated in a model of uterine LMS [209]. The predictive role of the structural epigenetic chromatin factor HMGA1 (high-mobility group protein A1) was demonstrated for chemosensitivity of LMS to trabectedin. Moreover, inhibition of HMGA1 with the mTOR inhibitor rapamycin restored the sensitivity of LMS cells for trabectedin in vitro and in vivo [210].

Several clinical trials have also been described. Recently, a phase I/II clinical trial of belinostat, a HDAC inhibitor, in combination with doxorubicin in advanced STS, was described with a moderate response; one patient with LMS reached a complete response [211]. Histone demethylases also could be overexpressed and provide the therapeutic option. Thus, a phase I dose-escalation study of zavondemstat (TACH101), an epigenetic targeting inhibitor of KDM4 histone demethylase, demonstrated the achievement of stable disease in one patient with LMS [212]. 

Novel therapeutic approaches of LMS treatment are summarized in Table 3.

## 5. Conclusions

In conclusion, this review has highlighted the complex interplay between signaling and epigenetic alterations in LMS pathogenesis. Recurrent mutations in genes, such as *TP53* and *RB1*, along with widespread epigenetic dysregulation, including DNA methylation and histone modification changes, contribute to the disruption of critical signaling changes, notably in the DNA damage and repair system, PI3K/AKT/mTOR and RAS/MAPK cascades, and epigenetic regulators.

Most of the strategies under consideration, including the modulation of the Hedgehog, Wnt/β-catenin, and kinase pathways, as well as individual epigenetic regulators, are currently based exclusively on preclinical in vitro and in vivo models. Although these approaches provide a theoretical foundation, they lack direct translational value until confirmed in clinical trials. Among the directions reviewed, inhibitors of the mTOR/PI3K and Notch pathways, immune checkpoint blockers, eribulin-containing regimens, and epigenetic modulators have reached the stage of clinical evaluation. However, all studies on these approaches were conducted in small cohorts, which does not allow the results to be considered sufficient for implementation into routine practice.

The most thoroughly studied and promising approach among those mentioned in the article is the combination of olaparib with agents already used for leiomyosarcoma. Although the phase II/III trial did not meet its primary endpoint of progression-free survival, some patients experienced durable and stable responses. According to the authors, this suggests that the strategy may remain viable for a carefully selected subgroup of patients—for example, those with tumors characterized by a high PARP1 expression.

Overall, all the described approaches face common limitations, such as leiomyosarcoma heterogeneity, the lack of validated predictive biomarkers for most targets (which hinders personalized therapy), and small sample sizes in clinical studies, limiting the statistical power and generalizability of the results.

The development of diagnostic techniques ex vivo and in vitro, including chemoresistance assays and liquid biopsy methods for real-time monitoring and the stratification of patients based on molecular subtypes and the integration of genomic and transcriptomic data in treatment planning, may be useful for the replication of the complex molecular and microenvironmental landscape of LMS, as well as for the further selection of an optimal treatment, potentially improving therapeutic strategies and patient outcomes.

## Figures and Tables

**Table 1:** Characteristics of LMS molecular subtypes.

LMS Molecular Subtype	Cell Phenotype	Molecular Findings and Associated Signaling	References
Well-differentiated soft-tissue LMS	Vascular, gastrointestinal smooth muscle	Genes involved in smooth muscle function (*MYLK*, *MYH11*, *LMOD1*, *MYOCD*, *CALD1*, *DES*, *CNN1*, *ACTG2*, *SLMAP* and *CASQ2*); *TP53* mutations; Amplification of growth factor genes (*PDGFRA*, *LRRC15*, *IGF1R*).	[89,92]
Dedifferentiated soft-tissue LMS	Vascular smooth muscle	Translation, translational elongation, protein localization, epithelial-to-mesenchymal transition, and tumorigenesis (*CDK6*, *MAPK1,3* and *HOXA1* overexpression; *Akt* signaling gene overexpression); *TP53* and *RB1* mutations.	[89,92,93]
Uterine LMS	Vaginal smooth muscle; low collagen density	*PTEN* loss; Overexpression of *Akt* signaling genes; *TP53* mutations; *RB1* loss; Dystrophin loss; Metabolic process, ion transport, and regulation of transcription; Increased expression of collagen-remodeling genes (*MMP14*).	[89,94,95]

**Table 2:** MiRNAs involved in the pathogenesis of LMS.

MiRNA	Role in LMS	References
MiRNA-1	Tumor suppressor, suppressor of stemness regulator zinc finger transcription factor ZNF281	[120]
MiRNAs from let-7 family	Tumor suppressors, HMGA regulation	[112]
MiRNA-152	Tumor suppressor	[116,117]
MiRNA-93	Oncogene	[116,117]
MiRNA-106b	Oncogene	[116,117]
MiRNA-17-92	Oncogene	[116,117]
MiRNA-221	Oncogene	[116,117]
MiRNA-320a	Tumor suppressor	[116,117]
MiRNA-654-3p	Tumor suppressor	[20,109,110]
MiRNA-181b	Tumor suppressor	[110]
miRNA-34a	Tumor suppressor	[110]
miRNA-369-3p	Tumor suppressor	[110]
miRNA-10b-5p	Tumor suppressor	[113]
MiRNA-130b	Oncogene	[118]
miRNA-223	Tumor suppressor (MDM2-targeting)	[115]
miRNA-379	Tumor suppressor (MDM2-targeting)	[115]
miRNA-377	Tumor suppressor (MDM2-targeting)	[115]
miRNA-495	Tumor suppressor (MDM2-targeting)	[115]
miRNA-758	Tumor suppressor (MDM2-targeting)	[115]
miRNA-486-5p	Tumor suppressor (CDK4-targeting)	[115]
miRNA-539	Tumor suppressor (CDK4-targeting)	[115]
miRNA-200c	Tumor suppressor, EMT inhibition	[116,117]

**Table 3:** New therapeutic approaches in the treatment of LMS.

Approach/Molecular Target	Therapeutic Agents/Combinations	Development Stage	Degree of Evidence and Comments
DNA damage and repair pathways: ATR, BRCA1/2, PARP-1	Elimusertib; combinations of olaparib and cisplatin; temozolomide and olaparib; olaparib and trabectedin	Preclinical: ATR inhibitor in an ATRX-mutant LMS model in vivo; olaparib in a HRD model. Clinical: Phase II (temozolomide and olaparib), case reports, combination studies.	Conflicting data: Early phase II studies were encouraging, but subsequent studies failed to show a significant benefit in disease-free survival compared with standard therapy.
Hedgehog pathway: SMO, GLI	LDE225; GANT61; 5-aza-dC	Preclinical: in vivo/in vitro. Some SMO inhibitors are already FDA-approved for other tumors, but there are no clinical data specifically for LMS.	Preliminary, further study required.
Angiogenesis: VEGF and VEGFR, endoglin, CSF1 and CSF1R	PTK787; combinations of bevacizumab and valproic acid and axitinib, pembrolizumab	Preclinical: PTK787 in vitro. Clinical: Phase I/II bevacizumab and valproic acid; Phase II axitinib and pembrolizumab.	Clinical trials: Partial responses in LMS, manageable toxicity. No convincing phase III data.
Immunotherapy: PD-1 и PD-L1, MAGE-A4, CD70, CD47	Nivolumab and pembrolizumab	Clinical studies I/II PD-1/PD-L1; preclinical for CD70, CD47, and MAGE-A4.	Low efficacy of anti-PD-1/PD-L1 monotherapy without clear clinical benefit. Combination strategies are being studied.
Proliferative signals: MAP2K4, TYRO3/AXL, HGF/SF and c-Met, PLK4/ATM, ALK	PLX8725; HGF/SF:c-Met inhibitors; CFI-400945 and AZD0156; alectinib; lorlatinib	Mainly preclinical; clinical case for ALK inhibitors.	Preliminary, further study required. A 16-month response has been reported for ALK inhibitors in one clinical case.
Wnt/β-catenin pathway: β-catenin, TRPV4	Potential TRPV4 inhibitors	Preclinical: in vivo/in vitro.	Low; clinical significance not determined.
PI3K/AKT/mTOR pathway: pS6RP/p4EBP1, PI3KCB/*PTEN*, mTORC1/2	BEZ235; rapamycin/sirolimus and MLN8237; curcumin; EGCG; sirolimus and ganestespib; GSK2636771; ridaforolimus; sapanisertib and metformin	Preclinical and clinical: Phase I/II SARC023, phase II GSK2636771, phase III ridaforolimus, dose-escalation sapanisertib and metformin.	Clinical activity is modest. Ridaforolimus improved PFS but not OS in phase III. GSK2636771 showed moderate activity in *PTEN*-inactivated patients. Sapanisertib and metformin showed early activity in two patients with LMS.
Notch pathway: HES1	DAPT; MK-0752; LY900009; LY3039478	Preclinical: Phase I for LY900009 and LY3039478.	Early data: LY3039478 showed clinical activity (necrosis, metabolic response, tumor shrinkage) in patients with LMS, including dose/regimenting for phase II.
Other signaling pathways and markers: Bcl-2, hENT1, stathmin, TUBB3, Hippo, PPARγ, PR, RNA polymerase I, γ-secretase	Eribulin and gemcitabine; eribulin and lenvatinib; NSC-260594/XMH95; CX-5461; MK-0752 and doxorubicin/docetaxel/gemcitabine; pioglitazone; ulipristal acetate; melatonin	Phase II clinical trials: eribulin and gemcitabine. Phase Ib/II: eribulin and lenvatinib. The rest are preclinical in vitro/in vivo/PDX studies.	Eribulin-containing combinations demonstrated clinical activity and a favorable safety profile. Other areas are preliminary.
Epigenetic regulators: BET (BRD2/3/4, BRD9), HDAC, DNMT, SUMOylation, HMGA1, KDM4	JQ1, TP-472, I-BET762; quisinostat and doxorubicin; azacitidine, 5-aza-dC, guadecitabine; proscillaridin A, lanatoside C; 2-D08; rapamycin for restoration of sensitivity to trabectedin; belinostat and doxorubicin; zavondemstat (TACH101)	Preclinical and clinical: Phase I/II belinostat and doxorubicin; phase I zavondemstat.	Preclinical activity is pronounced. Clinical case: A complete response in one patient with LMS with belinostat and doxorubicin; disease stabilization in one patient with LMS with zavondemstat.

## Data Availability

No new data were created or analyzed in this study. Data sharing is not applicable to this article.

## Abbreviations

The following abbreviations are used in this manuscript:

ACTB	Actin Beta
*ACTG2*	Actin Gamma 2
ADAR	Adenosine Deaminase Acting on RNA (gene family)
AGT	Angiotensinogen
ALK	Anaplastic Lymphoma Kinase
ALKBH5	AlkB Homolog 5, RNA Demethylase
ALX1	ALX Homeobox 1
APC	Adenomatous Polyposis Coli
AS1	ABHD11 Antisense RNA 1
ASS1	Argininosuccinate Synthetase 1
ATM	Ataxia Telangiectasia Mutated
ATR	ATR Serine/Threonine Kinase
ATRX	ATRX Chromatin Remodeler
AXL	AXL Receptor Tyrosine Kinase
BBC3	BCL2-Binding Component 3
BCL6	B-Cell Lymphoma 6
BCOR	BCL-6 Corepressor
BCORL	BCL-6 Corepressor Ligand
BER	Base Excision Repair
BET	Bromodomain and Extra-Terminal Domain (gene family)
BIK	BCL2-Interacting Killer
BMI1	B Lymphoma Mo-MLV Insertion Region 1 Homolog
BRAF	B-Raf Proto-Oncogene, Serine/Threonine Kinase
BRCA	Breast Cancer (gene family)
BRD	Bromodomain (gene family)
*CALD1*	Caldesmon 1
CARMN	Cardiac Mesoderm Enhancer-Associated Non-Coding RNA
CASP	Caspase (gene family)
*CASQ2*	Calsequestrin 2
CBLN1	Cerebellin-1
CCND	Cyclin D
CCNE	Cyclin E
CD	Cluster of Differentiation (gene family)
CDK	Cyclin-Dependent Kinase (gene family)
CDKN	Cyclin-Dependent Kinase Inhibitor (gene family)
CHEK	Checkpoint Kinase (gene family)
*CNN1*	Calponin 1
COMMD1	Copper Metabolism Domain Containing 1
CORIN	Corin, Serine Peptidase
CSF1	Colony-Stimulating Factor 1
CREBBP	Cyclic AMP Response Element-Binding Protein
CRYM	Crystallin Mu
CTA	Cancer-Testis Antigen
CTHRC1	Collagen Triple Helix Repeat Containing 1
CTNNB1	Catenin Beta 1
DAXX	Death-Associated Protein 6
DDR	DNA Damage and Repair System
DEFB132	Defensin Beta 132
*DES*	Desmin
DMNT	DNA Methyltransferase (gene family)
DUSP	Dual-Specificity Phosphatase (gene family)
4EBP1	Eukaryotic Translation Initiation Factor 4E-Binding Protein 1
EEF1A1	Eukaryotic Translation Elongation Factor 1 Alpha 1
EGCG	Epigallocatechin-3-gallate
EGFR	Epithelial Growth Factor (gene family)
EGR2	Early Growth Response 2
EMT	Epithelial–Mesenchymal Transition
EPC1	Enhancer of Polycomb Homolog 1
ER	Estrogen Receptor
ERBB2	ErbB-2 Receptor
ERK5	Extracellular Signal-Regulated Kinase 5
EZH2	Enhancer of Zeste 2 Polycomb Repressive Complex 2 Subunit
FANCA	Fanconi Anemia Complementation Group A
FGFR	Fibroblast Growth Factor Receptor (gene family)
FLT3	FMS-Like Tyrosine Kinase 3
FOLR3	Folate Receptor Gamma
FOXP1	Forkhead Box P1
FTO	Fat Mass and Obesity-Associated
GATA2	GATA-Binding Protein 2
GJA1	Gap Junction Protein Alpha 1
GLI	GLI Family Zinc Finger 1
γH2AX	Phosphorylated Form of a Histone Variant H2AX at Ser 139
HAT	Histone Acetyltransferase (gene family)
HDAC	Histone Deacetylase (gene family)
HDM	Histone Demethylase (gene family)
HES1	Hairy and Enhancer of Split 1
HGF/SF	Hepatocyte Growth Factor/Scatter Factor
HIST3H3	Histone H3
HMGA	High-Mobility Group Proteins Characterized by an AT-Hook
HMT	Histone Methyltransferase (gene family)
*HOXA1*	Homeobox A1
HRASLS	H-RAS-Like Suppressor
HRD	Homologous Recombination Deficiency
HRK	Harakiri, BCL2-Interacting Protein
IDO1	Indoleamine 2,3-Dioxygenase 1
IGF	Insulin-Like Growth Factor (gene family)
IGF1R	Insulin-Like Growth Factor 1 Receptor
IGLON5	Ig Family Containing LAMP, OBCAM, and NTM
IMP3	IMP U3 Small Nucleolar Ribonucleoprotein 3
ITD	Internal Tandem Duplication
JAK	Janus Kinase
JAZF1	JAZF Zinc Finger 1
KANK2	KN Motif and Ankyrin Repeat Domains 2
KANSL1	KAT8 Regulatory NSL Complex Subunit 1
KAT6B/A	K(Lysine) Acetyltransferase 6A/B
KDM	Histone Lysine Demethylase (gene family)
KIT	KIT Proto-Oncogene, Receptor Tyrosine Kinase
KMT	Histone Lysine Methyltransferase (gene family)
KRAS	KRAS Proto-Oncogene, Gtpase
LAG-3	Lymphocyte Activation Gene 3
LAMA4	Laminin Subunit Alpha 4
LMNA	Lamin A
*LMOD1*	Leiomodin 1
LMS	Leiomyosarcoma
LMP2	Latent Membrane Protein 2
lncRNA	Long Non-Coding RNA
LPP	LIM Domain Containing Preferred Translocation Partner in Lipoma
*LRRC15*	Leucine-Rich Repeat Containing 15
MAGE-A4	Melanoma-Associated Antigen 3, Family Member A4
MAN1A1	Mannosidase Alpha Class 1A Member 1
MAPK	Mitogen-Activated Protein Kinase
MASP1	MBL-Associated Serine Protease 1
MDM2	Mouse Double Minute 2
MGMT	O-6-Methylguanine-DNA Methyltransferase
MED12	Mediator Complex Subunit
MEF2C	Myocyte-Specific Enhancer Factor 2C
MEIS	Myeloid Ecotropic Viral Integration Site
MEN1	Menin 1
MET	MET Proto-Oncogene
METTL	Methyltransferase 5, N6-adenosine
MIF	Macrophage Migration Inhibitory Factor
MMP	Matrix Metallopeptidase (gene family)
MSH	Muts Homolog (gene family)
MSI	Microsatellite Instability
MTAP	Methylthioadenosine Phosphorylase
MTOR	Mechanistic Target Of Rapamycin Kinase
MYC	MYC Proto-Oncogene, Bhlh Transcription Factor
*MYH11*	Myosin Heavy Chain 11
*MYLK*	Myosin Light Chain Kinase
*MYOCD*	Myocardin
NER	Nucleotide Excision Repair
NKX6-1	NK6 Homeobox 1
NLGN	Neuroligin (gene family)
NNMT	Nicotinamide N-Methyltransferase
NOTCH	Notch Receptor
NOXA	NADPH Oxidase Activator 1
NPTX2	Neuronal Pentraxin 2
NR4A3	Nuclear Receptor Subfamily 4 Group A Member 3
NTRK	Neurotrophic Tropomyosin Receptor Kinase
ORM1	Orosomucoid 1
OSTN	Osteocrin
PART1	Prostate Androgen-Regulated Transcript 1
PBX3	Pre-B-Cell Leukemia Homeobox 3
PD-1	Programmed Cell Death Protein 1
PDGFR	Platelet-Derived Growth Factor Receptor
PD-L1	Programmed Cell Death Protein Ligand 1
PI3KCB	Phosphatidylinositol-4,5-Bisphosphate 3-Kinase Catalytic Subunit Beta
PI3K	Phosphatidylinositol-4,5-Bisphosphate 3-Kinase
PLAG1	Pleomorphic Adenoma Gene 1
PLK1	Polo-Like Kinase 1
PPARγ	Peroxisome Proliferator-Activated Receptor Gamma
PR	Progesterone Receptor
PRL	Prolactin
PSDM11	Proteasome 26S Subunit, Non-Atpase 11
*PTEN*	Phosphatase and Tensin Homolog
PUMA	P53 Upregulated Modulator Of Apoptosis
RAD51	RAD51 Recombinase
RAD51B	RAD51 Paralog B, DNA Repair Protein RAD51 Homolog 2
RAF1	RAF1 Proto-Oncogene
*RB1*	Retinoblastoma
RBPMS	RNA-Binding Protein with Multiple Splicing
ROR2	Receptor Tyrosine Kinase-Like Orphan Receptor 2
RORB	RAR-Related Orphan Receptor B
ROS1	ROS Proto-Oncogene 1, Receptor Tyrosine Kinase
S6RP	Ribosomal Protein S6
SATB1	SATB Homeobox 1
SETD7	SET Domain Containing 7, Histone Lysine Methyltransferase
SGK1	Serum/Glucocorticoid-Regulated Kinase 1
SHMT1	Serine Hydroxymethyltransferase 1
SLCO5A1	Solute Carrier Organic Anion Transporter Family Member 5A1
SLITRK4	SLIT- and NTRK-Like Family Member 4
*SLMAP*	Sarcolemma-Associated Protein
α-SMA	α-Smooth Muscle Actin
SMO	Smoothened
SRSF5	Serine- and Arginine-Rich Splicing Factor 5
STAT	Signal Transducer And Activator Of Transcription
STEAP4	Six Transmembrane Epithelial Antigen Of Prostate
STS	Soft-Tissue Sarcoma
SUV39H2	Histone H3K9 Methyltransferase
TACC1	Transforming Acidic Coiled-Coil Containing Protein 1
TCGA	The Cancer Genome Atlas
TCR	T-Cell Receptor
TDO2	Tryptophan 2,3-Dioxygenase
TELO2	Telomere Maintenance 2
TERT	Telomerase Reverse Transcriptase
THBS1	Thrombospondin-1
TIM-3	T-Cell Immunoglobulin Mucin 3
TIMP2	TIMP Metallopeptidase Inhibitor 2
TMB	Tumor Mutational Burden
TNFα	Tumor Necrosis Factor Alpha
TNNT1	Slow Skeletal Troponin T
TNS1	Tensin 1
TOP2A	Topoisomerase 2
TOPORS	TOP1-Binding Arginine/Serine-Rich Protein, E3 Ubiquitin Ligase
*TP53*	Tumor Protein P53
TP53BP1	Tumor Protein P53-Binding Protein 1
TPM3	Tropomyosin 3
TPR	Translocated Promoter Region, Nuclear Basket Protein
TSPAN	Tetraspanin (gene family)
TRIM13	Tripartite Motif Containing 13
TRPS1	Tricho-Rhino-Phalangeal Syndrome 1
TRPV4	Transient Receptor Potential Vanilloid 4
TUBB3	Tubulin Beta 3 Class III
TYRO3	TYRO3 Protein Tyrosine Kinase
UPA	Ulipristal Acetate
VEGF	Vascular Endothelial Growth Factor (gene family)
VIPR2	Vasoactive Intestinal Peptide Receptor 2
WNT	Wingless-Type MMTV Integration Site Family
WTAP	Wilms Tumor Suppressor Gene
XPO1	Exportin 1
XRCC3	X-Ray Repair Cross-Complementing Group 3
XRN2	5′-3′ Exoribonuclease 2
YTHDF1-3	YTH Domain Family 1-3
YWHAE	Tyrosine 3-Monooxygenase/Tryptophan 5-Monooxygenase Activation Protein Epsilon
ZC3H7	ZC3H7B Zinc Finger CCCH-Type Containing 7B
ZEB1/2	Zinc Finger E-Box-Binding Homeobox 1/2
ZFP36L1	ZFP36-Like 1 Zinc Finger CCCH-Type
